# Independent effects of statistical learning and top-down attention

**DOI:** 10.3758/s13414-020-02115-x

**Published:** 2020-09-09

**Authors:** Ya Gao, Jan Theeuwes

**Affiliations:** 1 Department of Experimental and Applied Psychology, Vrije Universiteit Amsterdam, Van der Boechorststraat 7, 1081 BT Amsterdam, The Netherlands; 2 Institute Brain and Behavior Amsterdam (iBBA), Amsterdam, the Netherlands

**Keywords:** Attentional capture, Visual search, Working memory, Statistical regularities, Top-down attention

## Abstract

It is well known that spatial attention can be directed in a top-down way to task-relevant locations in space. In addition, through visual statistical learning (VSL), attention can be biased towards relevant (target) locations and away from irrelevant (distractor) locations. The present study investigates the interaction between the explicit task-relevant, top-down attention and the lingering attentional biases due to VSL. We wanted to determine the contribution of each of these two processes to attentional selection. In the current study, participants performed a search task while keeping a location in spatial working memory. In Experiment 1, the target appeared more often in one location, and appeared less often in other location. In Experiment 2, a color singleton distractor was presented more often in location than in all other locations. The results show that when the search target matched the location that was kept in working memory, participants were much faster at responding to the search target than when it did not match, signifying top-down attentional selection. Independent of this top-down effect, we found a clear effect of VSL as responses were even faster when target (Experiment 1) or the distractor (Experiment 2) was presented at a more likely location in visual field. We conclude that attentional selection is driven by implicit biases due to statistical learning and by explicit top-down processing, each process individually and independently modulating the neural activity within the spatial priority map.

## Introduction

It is important to be able to direct our attention to those events that are relevant to us and prevent distraction by events that are unimportant. For example, when driving along a busy street, we have to attend to traffic signs, road markings, and look out for potential targets, such as pedestrians suddenly crossing the road. While doing so, we should ignore the buzzing sound of our telephone telling us that a new message came in and the neon flashing advertising lights of the stores along the road. Attentional selection is crucial for survival and entails the filtering and attenuation of incoming information (Broadbent, 1958).

Traditionally, attentional selection is claimed to be the interplay between competitive gains (Desimone & Duncan, 1995) that arise from strategic top-down processes consistent with our goals and intentions (Leber & Egeth, 2006) and bottom-up saliency driven processes that tend to bias attention towards objects that stand out from the environment (Theeuwes, 2010). Recently, however a new framework was presented that considers three separate factors that affect attentional selection. In addition to top-down and bottom-up selection, it was argued that lingering biases of previous selection episodes (i.e., selection history) plays an important role in attentional selection (Awh, Belopolsky, & Theeuwes, 2012; Failing & Theeuwes, 2018; Theeuwes, 2018, 2019). Critically, lingering biases, referred to as “selection history,” may drive attention selection towards particular objects that are neither part of the top-down set of the observer (i.e., objects that are irrelevant for the task) nor do they stand out from the environment to capture attention in a bottom-up way (Theeuwes, 2018).

Selection history plays an important role when there are particular statistical regularities present in the environment. It has been shown that visual statistical learning (VSL) of target and/or distractor locations has a large effect on attentional selection. VSL refers to the mechanism that enables observers to extract the distributional properties from sensory input across space and time (Frost, Armstrong, Siegelman, & Christiansen, 2015). Several studies have shown the effect of statistical regularities on attentional selection. For example, contextual cueing studies have shown that search for a target is more efficient when it appears reliably in specific locations within displays previously searched relative to when these targets appear at random locations within new displays (Chun & Jiang, 1998). Moreover, Geng and Behrmann (2005) showed that targets presented in high-probability locations are detected faster than those in low-probability locations (see also Ferrante et al., 2018; Jiang, Swallow, Rosenbaum, & Herzig, 2013c). Recently, Wang and Theeuwes (2018a, 2018b, 2018c; see also Ferrante et al., 2018; Goschy, Bakos, Müller, & Zehetleitner, 2014) showed VSL for distractor locations and demonstrated that locations that more often contain a distractor are suppressed relative to all other locations (Wang, van Driel, Ort, & Theeuwes, 2019). Overall, these findings are considered to be evidence that implicit statistical regularities that usually cannot be explicitly reported by the observer (see Ferrante et al., 2018; Wang & Theeuwes, 2018b) can bias attention such that locations that are likely to contain a target are enhanced and locations that are likely to contain a distractor are suppressed. Local spatial attentional enhancement and local spatial attentional inhibition determine the attentional priority of individual locations within priority maps of space (Theeuwes, 2018).

The notion that “selection history” (i.e., statistical learning), above and beyond top-down and bottom-up processes, as an important factor in attentional selection, is well established (Anderson, 2016; Awh et al., 2012; Chelazzi, Perlato, Santandrea, & Della Libera, 2013; Failing & Theeuwes, 2018; Theeuwes, 2018, 2019; Todd & Manaligod, 2018). Critically, however, many have argued that selection history effects should not be considered to be a separate category but instead should be considered to represent effects that are typically labelled as “top-down” (Egeth, 2018; Gaspelin & Luck, 2018; Navalpakkam & Itti, 2006; Sisk, Remington, & Jiang, 2018; Wolfe, Butcher, Lee, & Hyle, 2003). Indeed, some have argued that top-down attention should be used for anything that is affected by “context, learning, or expectation” (Gaspelin & Luck, 2018), while others have claimed that anything that is not driven by factors “outside” the organism (physical saliency of objects in the world) should by default be considered to be on “inside” the organism and therefore “top-down” (e.g., Egeth, 2018; Wolfe et al., 2003). Yet as argued before (Theeuwes, 2018), it is crucial to distinguish between selection driven by lingering “selection history” biases and selection that is truly top-down, volitional, and effortful. When one recognizes that these factors are different, one can study the interaction between these factors.

It is important to reiterate the differences between attentional top-down control and attentional biases due to statistical learning. Attentional biases due to statistical learning are typically assumed to be implicit, automatic, and often operate outside awareness. Learning resulting in these biases takes place even when top-down executive resources are fully occupied by additional tasks (Gao & Theeuwes, 2020). These biases due to VSL can result in the attentional enhancement or suppression of particular locations in space. On the other hand, attentional top-down control is in principle effortful and volitional representing conditions in which observers actively have to directing attention to a location in space (see Theeuwes, 2018, for a detailed discussion regarding top-down control). Typically, in an experimental paradigm that explores top-down attention, on each trial, observers are asked to direct attention to a location in space before the display comes on. For example, in Posner’s classic cueing tasks (Posner, Snyder, & Davidson, 1980), before display onset, observers receive a central symbolic cue (e.g., an arrow or a verbal instruction) pointing to a location to which observers should direct their attention. The typical finding is that observers are more accurate and faster when the target appears at the cued location than when it occurred at the noncued location (see also Theeuwes, 1989).

Another way to ensure that observers direct attention in an effortful way to a location in space is to ask observers each trial to memorize the location of an object presented somewhere in the display. Awh, Jonides, and Reuter-Lorenz (1998) showed that storing and holding a location in working memory is accomplished by shifting and holding spatial attention to that location in space until memory recall (see Theeuwes, Belopolsky, & Olivers, 2009, for a review; Theeuwes, Kramer, & Irwin, 2011). Brain imaging studies have shown that the brain areas recruited for directing top-down attention basically overlap with those used for keeping a location in memory (Awh & Jonides, 2001; Munneke, Heslenfeld, & Theeuwes, 2010). In the current study, we employed this method to ensure that on each trial spatial attention was directed in a top-down effortful way to a specific location in space.

The goal of the current study was to investigate the interactions between volitional, top-down control in which observers direct attention to a location in space from trial-to-trial and lingering biases from previous selection episodes (i.e., selection history). In his recent framework, Theeuwes (2019) speculated that top-down, bottom-up, and selection history effects could very well represent three factors that each independently act on the saliency map. If that is the case, we would expect that volitional top-down effects and lingering biases each have additive contributions to selection. In other words, if observers have learned that the target is more likely to appear at a specific location in space, and if observers have directed their attention to that very same location in space, then both effects should add up. However, if these effects operate on the same underlying mechanism (i.e., spatial attention), then it is expected that the effects should interact. Indeed, if attention is already directed to a location in space in a top-down fashion, there may be few additional attentional benefits from VSL lingering biases towards that location.

In the present study, we employed a visual task that was originally developed by Ferrante et al. (2018). The visual search display consisted of four elements presented equidistantly from one another (one per visual quadrant) along an imaginary circle. The elements either had all the same color (Experiment 1) or three had the same color and one had a different color (Experiment 2). Using this task, Ferrante et al. (2018) demonstrated statistical learning of target probabilities: Performance was better for targets presented at relatively high-probability locations, and performance was impaired for targets at relatively low-probability locations. We combined Ferrante et al.’s (2018) task with a spatial working memory task in which observers were required, on each trial, to direct attention to a specific location in space, which could coincide with either the high-probability target location, the low-probability target location, or a regular location. We examined the interaction between the benefit of directing attention in a top-down way to a location in space with the benefits (and costs) of learning the target probabilities.

Previous studies have shown that spatial probabilities about the target do result in an attentional bias as participants are faster to detect a target positioned in high-probability locations than in low-probability locations (Geng & Behrmann, 2002, 2005; Jiang, Swallow, & Rosenbaum, 2013b; Jiang, Swallow, Rosenbaum, et al., 2013c; Jiang, Swallow, Won, Cistera, & Rosenbaum, 2015). For example, Geng and Behrmann (2005) investigated the role of spatial probabilities learning in a conjunction search task that was combined with endogenous (i.e., an arrow pointing to the likely target location) and exogenous cuing (an onset flash near a target location). As in previous studies, spatial probability induced an implicit attentional bias such that targets presented at high-probability locations were detected faster than those in low-probability or random-probability locations. Critically, the facilitation due to probability cueing was additive with endogenous cueing and interacted with the salient exogenous cue. It was concluded that spatial probability and the endogenous cueing produced independent effects, suggesting at least some separation in processing. Others also reported additive effects of lingering biases due to selection history and explicit cueing (see also Stankevich & Geng, 2014).

In contrast to Geng and Behrmann (2005), Jiang, Swallow, and Rosenbaum (2013b) showed that probability cueing was basically eliminated when an endogenous (arrow) cue was introduced. For example, in their Experiment 5, participants first learned which quadrant was likely to contain a target establishing a strong attentional bias towards one of the quadrants. When during test session, an endogenous arrow cue was introduced, there was no evidence of any learned attentional bias anymore, and an effect was only found when the endogenous arrow cue happened to point to the quadrant for which a bias was acquired. It was concluded that endogenous cuing takes precedence over probability learning.

There were some differences between these studies. For example, in Jiang, Swallow, and Rosenbaum (2013b), the arrow pointed to a whole quadrant, while in Geng and Behrmann (2005), the arrow pointed to a specific likely target location. In both studies, however, the probabilities of trial-by-trial endogenous cueing and the overall probabilities of statistical learning were interrelated, making the effects of endogenous cueing and effects of statistical learning less clear-cut. For example, in Jiang, Swallow, and Rosenbaum (2013b), when the arrow happened to point to the quadrant for which participants had already acquired an attentional bias, a benefit was found. In a case like this, it is remains unclear whether the benefit is due to both endogenous cueing and/or the lingering bias. In the current study, this problem was addressed by disassociating top-down spatial attention (i.e., keeping a location in memory) from the probabilities involved in learning the regularities in the display.

In the current study, we examined how explicit task-relevant, top-down attention interacted with the lingering attentional biases due to VSL. In Experiment 1, we investigated lingering biases due to statistical learning of target location probabilities; in Experiment 2 we examined lingering biases due to statistical learning of probabilities of distractor locations.

## Experiment 1

**Fig. 1:**
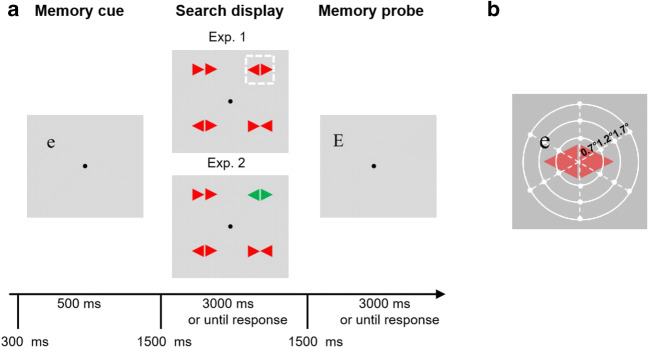
**a** Experimental procedure. Participants had to memorize the location of the letter *e*. After a 1,500-ms fixation display, a search display was presented. Participants were required to search for the target shape (the item with the two triangles pointing in the same [left or right] direction, its direction determined the response). The search display was presented for 3 s or until response. After a 1,500 ms blank, the memory probe (the letter *E*) appeared, and participants were required to indicate whether it was presented at the same or at a different location as the probe letter in the memory array. **b** Depiction of the dashed area in **a**; the white dots represent the 18 potential locations the memory cue *e* could appear in around the center of each search item in search display

In Experiment 1, one location had a high probability of containing the target (high prob: 37.5% of trials), and one location had a low probability of containing the target (low prob: 12.5%). The two other locations (labelled “intermediate”) had a 25% chance of containing the target. Unlike Ferrante et al. (2018), in this experiment, we did not include a singleton distractor (see Fig. 1). The location that needed to be memorized could match the target location (“matched-target”) or not match the target location (“unmatched”).

## Method

### Participants

G*Power software (Faul, Erdfelder, Lang, & Buchner, 2007) indicated that a sample of 32 participants would provide power of 0.8 to detect a significant, medium-sized (.25) within-subjects difference in response time under matched-target versus unmatched conditions. Thirty-six participants (32 females, *M*_age_ = 19.91 years, *SEM* = .33 years) were tested for course credit or payment of 8 euros. The study was approved by the Ethical Review Committee of The Faculty of behavioral and Movement Sciences of Vrije Universiteit Amsterdam. Participants had normal or corrected-to-normal vision and were naïve to the purpose of the experiment, and provided written informed consent before the experiment.

### Apparatus

Participants were tested in a dimly lit laboratory and held their chins on a fixed chin rest, 72 cm from the screen. The whole experimental procedure was written and controlled by OpenSesame (Version 3.2.8; Mathôt, Schreij, & Theeuwes, 2012) and run on an HP Compaq Pro 6300 SFF computer with a 22-in. liquid crystal display (LCD) color monitor (1,680 × 1,050-pixel resolution, 120-Hz refresh rate).

### Design

#### Visual search task

The search task was similar to that of Ferrante et al. (2018). Participants were required to search for a uniquely shaped item (target), and the location of this target followed an unequal probability distribution, which was unknown to participants. The visual search display consisted of four stimuli presented on an imaginary circle with a radius of 4°, centered around a central dot. Each stimulus was composed of two green (RGB: 0, 180, 0; luminance: 25 cd/m^2^) or red (220, 0, 0; 22 cd/m^2^) triangles (1°× 1°) presented on a light-gray background (186, 186, 186; 47 cd/m^2^). All these four items were of same color, either red or green. The target was the only item in the display with the two triangles pointing in the same direction (left or right), and the remaining three stimuli were pointing either outwardly or inwardly (with even probability).

In 37.5% of trials, the target in the search display appeared in one specific location only (high-probability location); in 12.5% of trials, the target appeared in another specific location (low-probability location), whereas in the remaining two locations (labelled “intermediate”), the target appeared with equal probability (25% per location; see Table 1). There were 12 possible combinations of choosing two from four high-probability and low-probability target locations, which were counterbalanced between participants.

**Table 1:** Spatial probability of memory cue and search stimuli in Experiments 1 and 2

Spatial probabilities (%)						
		Stimulus	Location			
			1	2	3	4
Exp. 1	Search task	Target	12.5	25	25	37.5
	Memory task	Memory cue	25	25	25	25
Exp. 2	Search task	Target	25	25	25	25
		Color-singleton	50	16.7	16.7	16.7
	Memory task	Memory cue	25	25	25	25

#### Working memory task

The task used here to guide attention in a top-down fashion to a specific location within the search display was like the task used by Awh et al. (1998). In each trial, before search, a memory display was presented containing a lowercase letter *e*, in 1.1° boldface, New York font (memory cue). After search, a test display was presented containing an uppercase letter *E* (memory probe). Participants were required to keep the location in memory and, after search, to determine whether the probe letter matched the location of the memory cue. Critically, the location of the letter kept in working memory overlapped with the location of the items that needed to be searched in the search task. We ensured that the exact location that needed to be stored in working memory was jittered around the location of the items of the search task. Specifically, there were 18 equidistantly potential locations on three imaginary concentric circles around the center of each search item, with the radius of 0.7°, 1.2°, and 1.7° (see Fig. 1b). The memory cue appeared around each search location with equal probability (25% per location), ensuring that there was no link between the memory task and search task. In test display, an uppercase letter *E* (memory probe), in 1° boldface, New York font was presented. The position of the memory probes with 50% probability matched the exact memorized location, with 25% probability that was 1.4° away from the memorized location (in six possible directions), and 25% probability that was 1.9° away from the memorized location (in six possible directions).

### Procedure

As shown in Fig. 1a, each trial started with a 300-ms fixation dot, followed by a memory cue for 500 ms. The search display appeared after a 1,500-ms interval after the offset of the memory display, and participants searched for the target stimuli, which showed the two triangles pointing in the same direction (left or right), and responded to the direction by pressing the *left* or *right* key. The test display appeared after a 1,500-ms interval after the offset of the search display, and participants needed to indicate whether the location of the memory probe was the same or was different by pressing the *s* (same) or *d* (different) key. The intertrial interval was between 500 and 750 ms at random. Participants were instructed to respond as soon and as accurately as possible in the search task, and as accurately as possible in the memory task. When participants made an error during the search or during the memory task, a buzzer sounded.

In total, eight blocks of 64 trials were run for each participant. Participants first completed a practice block of 24 trials with the visual search task only for targets appeared equally at each location. After each block, a feedback screen of mean accuracies and average response times (RTs) for two tasks was presented, and participants were asked to take a compulsory 1-minute break. When the experiment was finished, participants needed to indicate whether they had noticed any regularity regarding the location of the target, and irrespective of their answer had to indicate a location on the search display where they thought the target appeared more often and another location where the target appeared less often.

## Results

For the RT analyses, only trials with a correct response on both the search task and the memory task were included. Besides, trials in which the RTs were larger than 2.5 standard deviations from the average response time per condition per participant or less than 200 ms were excluded.

### Memory task

The mean accuracy for the memory task was 83.45%, significantly above chance, *t*(35) = 32.06, *p* < .001 (one-sample *t* test compared with 50%).

### Search task

**Fig. 2:**
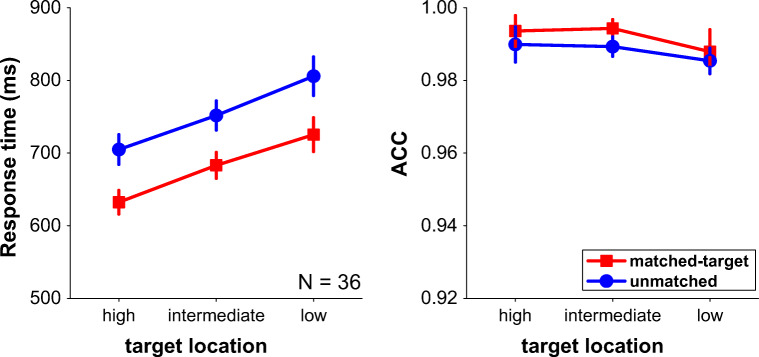
Mean response times (left panel) and mean accuracies (right panel) as a function of target at high, intermediate, and low-probability location for when the location in memory matched the target location (matched-target) or did not match the target location (unmatched). Error bars denote ±1 standard error of the mean.

We performed a 2 × 3 repeated-measures analysis of variance (ANOVA) on mean RT, with memory cue–target matched condition (matched-target, unmatched) and target location probability (high-probability location, intermediate location, low-probability location) as two within-subjects factors. Mean RTs and mean accuracies are presented in Fig. 2.

There was a large main effect on RT of the matched versus unmatched, *F*(1, 35) = 34.46, *p* < .001, η_p_^2^ = .50, indicating that when the location that is kept in working memory matched the location of the target during search task, participants were much faster (about 73.84 ms) than when the location kept in working memory did not match the location of the search target. This indicates that our spatial working memory task did recruit spatial attention, and suggests that during the whole search task, attention was directed in a top-down fashion to a specific location in space. In addition, there was also a large main effect of target location probability, *F*(2, 70) = 13.06, *p* < .001, η_p_^2^ = .27. Post hoc tests (LSD) indicated significant differences across all three conditions (*p*s < .01). Relative to the intermediate location probability, there were faster responses when target appeared at high-probability location and slower when it appeared at the low-probability location. Critically, there was no interaction between these two factors, *F*(2, 70) = .44, *p* = .65, η_p_^2^ = .01. The Bayesian statistics using JASP (JASP Team, 2018) showed strong evidence (BF_01_ = 11.13) that the data are better represented by two main effects model than the model that also includes the interaction. The absence of this interaction indicates that the effects of top-down spatial attention are independent of the effects of lingering attentional biases due to statistical learning. We conducted same analyses on mean accuracies, no significant main effect or interaction was found (*F*s < 2.73, *p*s > .11).

To determine the contribution of intertrial priming, we excluded all trials in which the target was presented at the same location as in the preceding trial and compared this with the original data which had target location repetitions. A 2 × 2 × 3 repeated-measures ANOVA on mean RTs, with repetition (with vs. without repeated trials), match condition (matched-target, unmatched), and target location (high-probability location, intermediate location, low-probability location) as three within-subjects factors, showed a reliable priming effect (main effect of repetition) *F*(1, 35) = 105.60, *p* < .001, η_p_^2^ = .751, and an interaction between repetition and target location, *F*(2, 70) = 4.50, *p* = .014, η_p_^2^ = .114.

To rule out the possibility that the statistical learning effect of target location is due to intertrial target location-based priming, we repeated the original ANOVA, excluding all trials in which the target location was repeated. The results basically remained the same, as there was still was a highly reliable effect of target location, *F*(2, 70) = 10.19, p < .001, η_p_^2^ = .23, which indicates that statistical learning occurs above and beyond intertrial priming.

### Awareness assessment

When asked whether they noticed anything regarding the regularities of the location of the target, 13 participants indicated that he or she noticed that there are some regularities regarding the distribution of the target, but only two of them reported the two correct locations. When forced to indicate which two locations contained the target more often and which location contained the target less often, these two participants and two other participants indicated the correct locations. When we excluded these four subject’s data and repeated the above analysis, all results remained the same. Overall, this analysis suggests that there is little evidence (if any) that participants were aware of the regularities.

## Discussion

The current findings are clear. There was a large effect of directing top-down attention to a location in space: When participants held a location in spatial working memory and this location matched the location where the target was in visual search, participants were much faster (about 73.84 ms) than when it did not match the location. This implies that attention was indeed directed to a location in space, giving rise to large cueing benefits (Posner, 1980). On top of this effect, there was an effect of statistical learning: when the target was presented at the high-probability location, participants were faster than at an intermediate probability location, and when presented at the low-probability location participants were slower than at the intermediate location. This latter effect of statistical learning of the target position is consistent with an earlier study that employed this very same statistical learning paradigm (Ferrante et al., 2018). It is important to note that there was perfect additivity between the effects of three levels of probability learning and two levels of top-down spatial attention. One possible explanation of finding perfect additivity (across three levels) is to assume that statistical learning and top-down attention affect different processes which are separated in time (Sternberg, 1969) even though alternative explanations are possible (McClelland, 1979). For example, according to a cascade model (Ashby, 1982; McClelland, 1979), multiple processes can operate continuously, providing partial output that can be used as input for the next process. According to such a cascade model, one can also find additive effects that do not necessarily imply the involvement of separate processes.

Note that these results cannot be explained by intertrial priming only. Even though there was intertrial priming, our analysis shows that when we take out all trials in which the target location was repeated, the effects remained basically the same. This is consistent with previous studies showing that intertrial priming cannot explain the effects of lingering biases due to statistical learning (Wang & Theeuwes, 2018a, 2018b, 2018c).

Our awareness measures show that most participants were not aware of the regularities that were present in the display, consistent with previous studies (Ferrante et al., 2018; Wang & Theeuwes, 2018a, 2018b, 2018c). If the aware participants were removed from the analysis, the effect remained the same.

## Experiment 2

In Experiment 2, we introduced a singleton distractor, similar to Ferrante et al.’s (2018). The singleton distractor was presented much more often in one location than in all other locations. It was shown before that this does result in suppression of the high-probability location relative to the low-probability location (Ferrante et al., 2018; Wang & Theeuwes, 2018a, 2018b, 2018c). The question was how top-down attention would interact with learned suppression.

## Method

### Participants

G*Power software (Faul et al., 2007) indicated that a sample of 28 participants would provide power of 0.8 to detect a significant, medium-sized (.25) within-subjects difference in response time under three memory cue matched conditions. Thirty-two participants (28 females, *M*_age_ = 20.15 years, *SEM* = .37 years) were tested for course credit or payment of 8 euros.

### Stimuli and apparatus

The stimuli and apparatus were the same as in Experiment 1, except that one of the distractors was a color singleton. We manipulated the probability distribution of distractor (color singleton) location. Among the four items in the visual search display, three were the same color (e.g., red), and the fourth was an alternative color (e.g., green), regarded as the color-singleton distractor. The target was the only item with the two triangles pointing in the same direction (left or right), and the distractor triangles were pointing either outwardly or inwardly (with even probability). The remaining two stimuli (nontargets or distractors) were always the same color as the target, and with one triangle pointing outwardly and one pointing inwardly. In the search task, participants needed to search for the target. The color-singleton distractor appeared in one location 50% of the time (high-probability location) and in the other (low probability) locations about 16.7% of the time. The target was equally likely to appear in all locations (25%; see Table 1). The high-probability location was counterbalanced between participants.

### Procedure

The procedure was identical to that of Experiment 1 (see Fig. 1), as participants also needed to complete both search task and memory task. In Experiment 2, participants first completed a practice block of 24 trials, with the visual search task for distractors appearing equally at each location, and then received five blocks of 96 trials in the formal experiment, in which the distractor appeared more often in one location than in all locations. Participants also needed to answer two questions after the whole experiment: They should report whether there was any regularity regarding the location of distractor, and irrespective of the answer had to indicate the location on the search display where they thought the distractor appeared more often.

## Results

### Memory task

The mean accuracy for the memory task was 82.14%, significantly above chance, *t*(31) = 30.92, *p* < .001 (one-sample *t* test compared with 50%).

### Search task

Distractor at high-probability versus low-probability distractor location

**Fig. 3:**
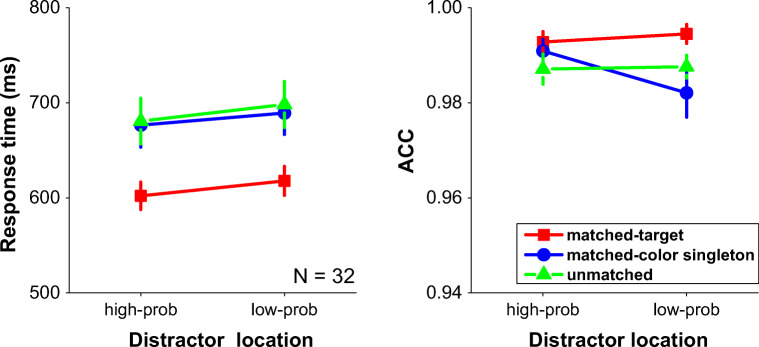
Mean response times (left panel) and the mean accuracies (right panel) as a function of distractor at high-probability versus low-probability location for when the location in memory matched the target location (matched-target), matched the color distractor location (matched-distractor) or match neither the target nor the color singleton distractor location (unmatched). Error bars denote ±1 standard error of the mean

We performed a 3 × 2 repeated-measures ANOVA on mean RTs and mean accuracies, with memory cue matched condition (matched-target, matched-color singleton, unmatched) and distractor location (distractor at high-probability location, distractor at low-probability location) as two within-subjects factors. Mean RTs and mean accuracies are presented in Fig. 3.

There was a significant main effect of matched condition, *F*(2, 62) = 25.31, *p* < .001, η_p_^2^ = .45, post hoc tests (LSD) indicated that participants responded remarkably faster (around 76.23 ms) when targets matched the memorized location than when the color singleton distractor or other items matched the location (*p*s < .001 in pairwise comparisons). There was no reliable difference when the memory cue matched the color distractor location or the other locations (*p* = .11). The main effect of distractor probability was also significant, *F*(1, 31) = 4.27, *p* < .05, η_p_^2^ = .12. RTs were generally faster when the color distractor was presented at high-probability location relative to the low-probability location. Consistent with the findings of Wang and Theeuwes (2018a, 2018b, 2018c) and Ferrante et al. (2018), there was suppression of the high-probability distractor location as capture was reduced for the high-probability relative to the low-probability locations. There was no reliable interaction between these two factors, *F*(2, 62) = .28, *p* = .76, η_p_^2^ = .01. The Bayesian statistics also supported the two main effects model 10.3 times more than the model plus the interaction. The absence of this interaction indicates that even though top-down spatial attention was allocated to a location in space, the statistical regularity of the probability of the color distractor singleton still had a relatively reliable effect on selection.

To rule out the possibility that this suppression effect for distractors presented at the high-probability versus low-probability location was due to intertrial location-based suppression (priming) effect, we excluded all trials in which the location of the distractor was repeated from one trial to the next. Then we compared this part of results with the original data that contained the repeated trials, and conducted a 2 × 3 × 2 repeated-measures ANOVA on mean RTs, with repetition (with repeated trials vs. without repeated trials), match condition (cue matched target location, cue matched distractor location, unmatched), and distractor location (distractor at high-probability location, distractor at low-probability location) as three within-subjects factors. We only found a main effect of match condition, *F*(2, 62) = 24.50, *p* < .001, η_p_^2^ = .44, and the marginal effect of distractor location, *F*(1, 31) = 3.99, *p* = .055, η_p_^2^ = .11. There was no effect of repetition, *F*(1, 31) = 1.45, *p* = .28, nor any interaction with the other variables (*p*s > .46), which indicates that statistical learning occurs above and beyond intertrial priming.

We found participants made less errors when target at memorized location than distractor or other stimuli, F(2, 62) = 3.46, p = .037, η^2^_*p*_ = .10. Post-hoc tests (LSD) indicated both significant differences when distractor (p = .03) or other stimuli (p = .02) at memorized location, compared with the condition that target at memorized location. In addition, no distractor location main effect (F(1, 31) = 1.49, p = .23) nor the interaction (F(2, 62) = 2.33, p = .10) was reliable.

#### Target at high-probability versus low-probability distractor location

To measure the indirect statistical learning effect on target selection, we performed a 3 × 2 repeated-measures ANOVA on mean RTs and mean accuracies, with memory cue matched condition (matched-target, matched-color singleton, unmatched) and target location (target at high-probability distractor location, target at low-probability distractor location) as two within-subjects factors.

**Fig. 4.**
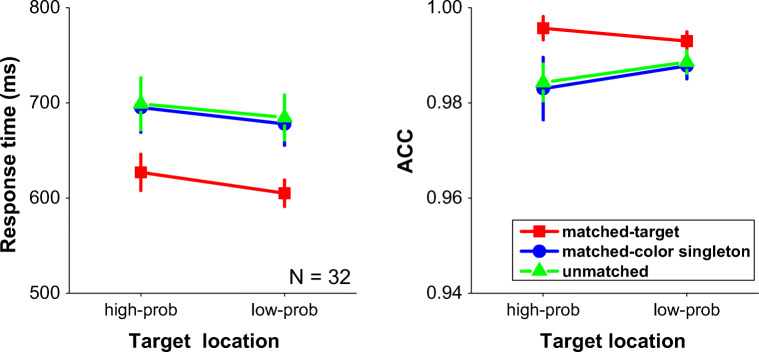
Mean response times (left panel) and the mean accuracies (right panel) as a function of target at high-probability distractor location versus low-probability distractor location for when the location in memory matched the target location (matched-target), matched the color distractor location (matched-distractor), or did not match either the target or the color singleton distractor location (unmatched). Error bars denote ±1 standard error of the mean.

As shown in Fig. 4, the main effect of matched condition was significant, *F*(2, 62) = 21.74, *p* < .001, η^2^_*p*_ = .41, Post-hoc tests (LSD) showed significant differences when the target was presented at the memorized location relative to when the distractor singleton or other elements were presented there (*p*s < .001). The main effect of target location probability was not reliable, *F*(1, 31) = 1.52, *p* = .23, η_p_^2^ = .05. There was no significant interaction between these two factors, *F*(2, 62) = .36, *p* = .70, η_p_^2^ = .01.

Accuracies basically followed RTs, the main effect of matched condition was significant, *F*(2, 62) = 3.45, *p* = .04, η_p_^2^ = .10. No target location main effect, *F*(1, 31) = .61, *p* = .44, or interaction, *F*(2, 62) = 1.22, *p* = .30, were significant.

### Awareness assessment

Five participants reported noticing that some locations contained the distractor more often than other locations, but none of them reported the correct location. When forced to indicate which location, another five participants indicated the correct high-probability distractor location. We excluded the data from these five participants who correctly reported the location and repeated the above analysis; all results were replicated except the main effect of distractor location, *F*(1, 26) = 2.82, *p* = .10, η_p_^2^ = .10.

## Discussion

The results show again that when participants held a location in spatial working memory and this location matched the visual search target location, participants were about 76.23 ms faster than when it did not match the location. Keeping a location in spatial working memory acts like top-down spatial attention (Awh et al., 1998; Theeuwes et al., 2011), which adds benefits for processing stimuli presented at that location (Posner, 1980). In addition to this effect, there was an effect of statistical learning: When the singleton distractor was presented at a high-probability location, there was less attentional capture than when this same color singleton was presented at a low-probability location, irrespective of where top-down spatial attention was directed. Again, there was no interaction between spatial top-down attention and lingering biases due to statistical learning, which indicates that top-down attention and statistical learning have independent effects on attentional selection.

Unlike in previous studies (Wang & Theeuwes, 2018a, 2018b, 2018c), in the current experiment, time to respond to the target when presented at the high-probability distractor location was not different than when the target was presented at any of low-probability locations (see Fig. 4). Even though inconsistent with Wang and Theeuwes (2018a, 2018b, 2018c), who used the additional singleton task, the current findings are in line with Ferrante et al. (2018), who used a paradigm comparable with the one used here. Ferrante et al. (2018) also showed no effect of the distractor probability manipulation on target selection processes. They speculated that this was due to an asymmetry between two forms of statistical learning, with statistical learning of the distractor location producing effects that were weaker than effects resulting from statistical learning of the target location.

As in Experiment 1, intertrial priming did not play a large role, and neither did awareness. When removing data from the five participants that showed some awareness of the statistical regularities, the effects became less robust, but likely due to diminished power. In any event, awareness does not play a large role in obtaining these effects (Ferrante et al., 2018).

## General discussion

In two experiments, we showed that the effect of directing spatial attention to a location in space and the effect of lingering biases due to statistical learning have additive effects on attentional selection. Additivity may suggest that these two factors independently affect two distinct selection stages that are separated in time (Sternberg, 1969). This may imply that due to implicit statistical learning, a priority map (a landscape) emerges with activation (priority gain) and suppression (priority loss) across the different locations. On top of this map, volitional top-down spatial attention may add an additional, independent priority gain for the location to which attention is directed. It implies that these two factors independently contribute to attentional selection. Note, however, that instead of assuming separate processing stages that are separated in time, there are alternative explanations for finding additivity. If one assumes a cascade model (McClelland, 1979) in which multiple processes operate simultaneously, it is possible that top-down attention does interact with the shaping of the priority map due to statistical learning.

The effects of statistical learning of regularities present in the visual field, as studied here, is also known under the term *spatial probability cueing* (Geng & Behrmann, 2002, 2005; Jiang, Swallow, & Rosenbaum, 2013b; Jiang, Swallow, Rosenbaum, et al., 2013c; Miller, 1988; Shaw & Shaw, 1977; Walthew & Gilchrist, 2006). In Shaw and Shaw’s (1977) classic study, participants had to search for a target letter appearing in eight different locations. Some locations were more likely to contain a target than others. The results indicated that participants were faster at finding those targets when they appeared in high-probability locations relative to low-probability locations. Similarly, Geng and Behrmann (2002) showed that participants were biased to search particular display regions that were more likely to contain a target than when all regions were equally likely to contain a target. Similarly, if a region was less likely to contain a target, attention was biased away from this region. Jiang, Swallow, and Rosenbaum (2013b) showed similar effects (see also Jiang, Swallow, & Capistrano, 2013a; Jiang, Swallow, Rosenbaum, et al., 2013c; Jiang et al., 2015): When the target was more likely to be present in one of the four quadrants, participants were faster to detect the target than when it was presented in a low-probability region. Recently, through statistical learning it was shown that participants can learn to suppress the location that is likely to contain a distractor (Failing, Wang, & Theeuwes, 2019; Wang & Theeuwes, 2018a, 2018b, 2018c).

Ferrante et al. (2018) demonstrated in the same paradigm we have used here that participants can learn to enhance locations that are likely to contain a target, and at the same time suppress locations that are likely to contain a distractor. Ferrante et al. (2018) concluded that statistical learning induced plasticity in the spatial priority map. Specifically, it is assumed that statistical learning changes the weights (both enhancement and suppression) within one spatial priority map, determining selection in a winner-take-all scenario. At any moment in time, the level of activity within this map determines which location is selected for further processing. The current findings suggest that in addition to statistical learning that determines the weights (pluses and minus) with this spatial priority map, there is another map in which top-down attention can boost (probably not suppress) selection in an additive way, above and beyond the weights set by statistical learning. There is strong evidence that top-down spatial attention can enhance the processing of items presented at a location to which attention is directed (Posner, 1980; Theeuwes & Van der Burg, 2011), while the evidence for top-down suppression is less clear-cut (see for Wang & Theeuwes, 2018a).

Unlike previous studies, we have used spatial working memory to direct spatial attention in a top-down fashion to a location in space. Usually, in almost all studies on top-down attention, the Posner arrow cue, with a particular validity (e.g., in 80% of trials it points to the likely target location), is used to direct attention in a volitional way. The advantage of using spatial working memory as we did in the current study is that the cue is always 100% valid (i.e., this is the location that participants had to memorize), and the working memory performance (tested after each search trial) indicates whether participants did in fact keep this location in memory. It is likely that with this method there is a stronger focus of attention to a location in space than with a traditional Posner arrow cue, possibly explaining why we obtained such large effects of top-down cueing (about 80 ms).

Our conclusion that the effects of lingering biases due to statistical learning are independent from the effects of top-down spatial attention are similar to the conclusions of Geng and Behrmann (2005), who examined the effect of target probabilities and endogenous and exogenous cueing in conjunction search. The endogenous cue consisted of an arrow pointing to the likely target location. The exogenous cue was a brief flash near a possible target location. The results with respect to the endogenous arrow cue are similar to the current finding, as the facilitation due to probability cueing was independent of the effect of endogenous cueing. Interesting, exogenous cueing interacted with probability cueing. On the basis of this study, it was concluded that spatial probabilities concerning the target location constitute a potent bias of visual processing representing an attentional cue that differs from endogenous and exogenous cueing.

Instead of considering these spatial probabilities as a form of cueing as conceived by Geng and Behrmann (2005), we interpret effects in terms of our model (Theeuwes, 2018, 2019), in which it is assumed that attentional selection is the result of the interaction between top-down, bottom-up, and selection history factors. Target and distractor probabilities affect selection history as participants learn the regularities in the display. Due to statistical learning, weights with the spatial priority map are set such that locations likely to contain a target are enhanced, and locations that are less likely to contain a target or are likely to contain a distractor are inhibited. This learning occurs implicitly and automatically, and participants have little, if any, explicit knowledge about these statistical contingencies (Ferrante et al., 2018; Wang & Theeuwes, 2018b). Due to this implicit statistical learning, a priority map (a landscape) emerges, with activation (priority gain) and suppression (priority loss) across the different locations. On top of this priority map, there is an intentional and effortful top-down direction of spatial attention that may result in priority gain for this one location similar to the “spotlight of attention” theory (Posner, 1980). Directing this spotlight is very much under volitional control, and participants are very much aware to which location they directed their attention. Unlike the implicit effect of statistical learning, top-down attention is very much explicit. These implicit and explicit effects seem to independently drive attentional selection.

One may further speculate how attentional selection is driven by these explicit top-down processing and implicit statistical learning. It is feasible that because of statistical learning, a prioritized landscape of neural activity emerges (Zelinsky & Bisley, 2015). On top of this landscape, there is the top-down “attentional spotlight” that serves to increase the gain for objects falling within the spotlight. The “spotlight” is typically associated with effortful shifting attention from location to the next (even though in the current study shifting attention was not needed).

Recently, Jiang (2018) described a dual-system model for spatial attention that somewhat resembles what we propose here. According to this model, “where” participants attend may be set up as a spatial priority map, consisting of spatial hot spots that are assigned with greater priority. It is assumed that these hot spots are modeled as a baseline shift in neurons coding different locations. In Jiang’s (2018) conception, this baseline shift is the result of bottom-up and top-down attention, whereas we claim that these baseline shifts are the results of statistical learning. In addition to the “where” component, Jiang (2018) described the “how” component as “shifts of spatial attention from one location to another,” which we would like to label as top-down spatial attention (similar to Posner’s spotlight of attention), while in Jiang’s (2018) model it is associated with statistical learning. So even though the components are similar, the implementation of this model is different, as we assume that statistical learning creates an implicit landscape of activations and inhibitions on which top-down spatial attention may operate.

This notion above could also explain why some have argued that attention cannot be split over multiple locations (i.e., the unitary spotlight of attention), while others claimed that multiple, spatially distinct regions of space can be selected at once, while ignoring in intervening regions (Awh & Pashler, 2000; Shaw & Shaw, 1977). It is feasible that the unitary spotlight of attention is related to what we have labeled here as explicit top-down processing, while the enhanced processing of multiple locations is related to implicit statistical learning shaping a landscape of neural activity.

In sum, the current study shows that there are two processes separately contributing to attentional selection. One the one hand, there is the explicit, top-down spatial enhancement of the processing of a location in space, and on the other hand, there are implicit and automatic lingering attentional biases (enhancement or suppression) due to VSL. Critically these processes seem to independently contribute to attentional selection.
